# Self-perceived quality of life, cognitive and behavioural impairment in amyotrophic lateral sclerosis

**DOI:** 10.1007/s00415-024-12639-z

**Published:** 2024-08-28

**Authors:** Ratko Radakovic, Amy Carroll, Alair Altiero, Carrie Reichwein, Susan Walsh, Elaine Niven, Sharon Abrahams, Zachary Simmons

**Affiliations:** 1 Department of Clinical Psychology and Psychological Therapies, Norwich Medical School, University of East Anglia, Norwich, UK; 2 Euan MacDonald Centre for Motor Neuron Disease Research, University of Edinburgh, Edinburgh, UK; 3 Alzheimer Scotland Dementia Research Centre, University of Edinburgh, Edinburgh, UK; 4 Cambridgeshire and Peterborough NHS Foundation Trust, Cambridge, UK; 5 Norfolk and Norwich University Hospital, Norwich, UK; 6 Department of Neurology, Pennsylvania State University, Hershey, PA USA; 7 Human Cognitive Neuroscience-Psychology, School of Philosophy, Psychology and Language Science, University of Edinburgh, Edinburgh, UK; 8 Psychology, School of Humanities, Social Sciences and Law, University of Dundee, Dundee, DD1 4HN UK

**Keywords:** Quality of life, behaviour, cognition, amytrophic lateral sclerosis

## Abstract

**Background:**

Self-perceived quality of life (QoL) is important in amyotrophic lateral sclerosis (ALS). Although caregiver burden and strain have been related to cognitive and behavioural impairment, there has been no comprehensive research looking at these impairments and how they may influence self-perceived QoL subdomains.

**Aims:**

To explore how cognitive and behavioural impairment are related to different areas of self-perceived QoL using disease-specific measures.

**Methods:**

This was a quantitative, cross-sectional, observational cohort study, utilising existing specialist ALS clinic data. Clinical and demographic variables were available as well as multidimensional measures, ALS-specific QoL Short Form (ALSsQoL-SF) results and the data from the Edinburgh Cognitive and Behavioural ALS Screen (ECAS). Group comparison and regression analyses were performed.

**Results:**

Data from 121 participants with ALS were analysed. 61.2% (*N* = 74) had either cognitive and/or behavioural impairment, with 28.9% (*N* = 35) with cognitive impairment (ALSci), 14.1% (*N* = 17) with behavioural impairment (ALSbi) and 18.2% (*N* = 22) with both (ALScbi). 38.8% (*N* = 47) were classified as having no impairments (ALSni). Those with ALSbi had significantly lower QoL in the domains of negative emotions and the interaction with people and the environment compared to those with ALSci and ALSni (*p*s < 0.05). Further, those with ALScbi had significantly lower QoL in the intimacy domains than those with ALSci and ALSni (*ps* < 0.05). Regression analysis showed specific cognitive and behavioural (inclusive of psychosis) predictors associated with specific QoL subdomains.

**Conclusions:**

Behavioural impairments effect QoL in specific subdomains, namely relating to internalising (negative emotions) and externalising (interaction with people and the environment subdomains, intimacy).

**Supplementary Information:**

The online version contains supplementary material available at 10.1007/s00415-024-12639-z.

## Introduction

Motor neuron disease (MND) is a terminal, neurodegenerative condition impacting physical function that progresses through different clinical stages, typified by upper and lower limb weakness, bulbar dysfunction (i.e. problems with speaking, swallowing or salivation), and progressive respiratory failure [1, 2]. As well as physical and functional deterioration, cognitive and behavioural impairments are well documented as occurring in amyotrophic lateral sclerosis (ALS), the most common form of MND [3–6]. These can be categorised using the ALS–frontotemporal spectrum disorder (ALS–FTSD) diagnostic criteria [7], which classifies these as of cognitive impairment (ALSci), behavioural impairment (ALSbi), both impairments (ALScbi), FTD (ALS–FTD) and no such impairments (ALSni). These impairments have been found to be variable across different clinical disease stages, with an emphasis on executive dysfunction across stages [8]. More recent research has found that, disease-specific and common impairments, such as apathy, disinhibition, executive dysfunction, and language problems have been observed across different clinical disease stages [9]. Furthermore, cognitive and behavioural impairments in ALS have been associated with difficulties with activities of daily living and increased caregiver burden or strain [10, 11].

There has been an increased focus on disease-specific QoL based on domains that are perceived as important by people with ALS (pwALS) relative to their everyday life in the context of the condition [12]. These are assessed by measures such as the ALS-specific QoL (ALSsQoL) [13, 14] instrument or the ALS Assessment Questionnaire-40 [15]. Previous research has documented that QoL in ALS can be associated with physical decline, pain, and fatigue [16–19]. Additionally, loss of bulbar function can make QoL worse, through difficulty in speaking and problems with salivation [20]. Mental health problems (anxiety and low mood or depression) have been associated with lower QoL [21, 22]. Further research has found that other factors such as spirituality and wider religious belief have been observed as a source of support in terms of QoL, extending beyond health-related QoL [23, 24]. Moreover, interaction with the environment and other people has also been noted as an important QoL domain, as a surrogate to feeling supported and having satisfying relationships to those close to the pwALS [25, 26]. Parallel to this, intimacy (physical and emotional) has also been observed as an important determinant or subdomain of QoL, particularly impacting emotional well-being for pwALS [27, 28]. This showcases the intricacy and multifaceted nature of QoL for pwALS. While previous research has explored QoL in relation to cognitive and behavioural impairment, findings have been variable in quality and mixed in their findings [29, 30], with a suggestion of behavioural impairment impacting QoL in MND [31]. The relationships between ALS-specific QoL subdomains and cognitive and behavioural impairments have not been explored.

As such, the primary research question sought to determine what the relationship between cognitive and/or behaviour impairment and self-perceived QoL for pwALS, using disease specific measures. The secondary research question, looked to explore if specific cognitive or behavioural impairments were associated with specific domains of self-perceived QoL. Guided by previous literature relative to mental health, QoL, caregiver strain and cognitive or behavioural problems in ALS, the prediction is that cognitive and behavioural impairment will negatively impact overall QoL, as well as mental health QoL subdomains (anxiety, depression) and those that associate with relational or systemic aspects of QoL, such as interacting with others and the environment.

## Methodology

### Design

This was a quantitative, cross-sectional, observational cohort study. The data available was secondary retrospective data, collected from 2014 to 2019 through medical record review from an ALS specialist clinic in the United States of America (USA).

### Participants

The dataset was composed of 135 pwALS from urban and rural areas of Pennsylvania, USA. Inclusion criteria were pwALS who were at least 18 years of age, were English speakers, had a diagnosis of familial or sporadic ALS using the revised El Escorial criteria [32], and had a cognitive assessment completed as a part of their standard care. Exclusion criteria were co-existing neurological or psychiatric illness (e.g. epilepsy, acquired brain injury, schizophrenia) or severe fatigue that would interfere with the individual’s ability to complete a cognitive assessment.

### Measures

#### Descriptive and clinical variables

Demographic information of age (years), sex (male/female), handedness (right/left/ambidextrous), and education (years) were available for pwALS. In terms of clinical information, comorbid FTD diagnosis (yes/no), site of onset (limb/bulbar), self-reported symptom onset date, and diagnosis date derived from medical records were also available.

The ALS Functional Rating Scale-Revised (ALSFRS-R) [33] is a self-rated, 12-items scale exploring severity of functional disability in ALS. Each item is scored on a 5-point Likert scale from 0 (poor functioning) to 4 (normal functioning) across four domains: bulbar function (items 1–3) fine motor (items 4–6), gross motor (items 7–9), and respiratory function (items 10–12). Total scores range from 0 (worst functioning) to 48 (normal functioning). The four domain scores were also calculated, ranging from 0 (worst functioning) to 12 (normal functioning) for each domain. The total score ranged from 0 (worst functioning) to 48 (normal functioning). The ALSFRS-R has been found to have an acceptable internal consistency reliability (Cronbach’s standardised alpha = 0.71), and has been validated against relevant physical and health status measures (i.e. predicted forced vital capacity, sickness impact profile).

The King’s clinical stages were also determined for by counting up the number of neurological regions involved by the disease (bulbar, upper limb, lower limb, respiratory/nutritional problems, Stage 1 = one region, Stage 2 = two regions, Stage 3 = three regions, and Stage 4 = respiratory/nutritional involvement) [2, 34]. These were estimated using the ALSFRS-R item level scores using an available algorithm for conversion to King’s clinical stages, a calculation that has demonstrated a strong correlation with actual King’s clinical stage (correlation coefficient = 0.95) [1].

#### Cognitive and behavioural functioning assessment

The Edinburgh Cognitive and Behavioural ALS Screen (ECAS) [35] is a 15–20 min, multidomain neuropsychological assessment quantifying cognitive and behavioural functioning developed specifically for ALS. It is composed of a cognitive assessment and behavioural interview. The cognitive assessment examines three ALS-specific domains (verbal fluency, executive functioning including social cognition, language) and two ALS-nonspecific domains (memory and visuospatial), providing a total score that can range from 0 (most impaired) to 136 (most intact). Published cutoffs are available, with a total score of less than or equal to 105 and/or an ALS-specific score of less than or equal to 77 indicating cognitive impairment [36]. The ECAS was found to have an acceptable internal consistency reliability (Cronbach’s standardised alpha = 0.77) and has been validated against a full neuropsychological assessment battery, showing 85% sensitivity and 85% specificity for detecting cognitive impairment using the ECAS total and ALS-specific scores [37].

The behaviour interview is composed of ten items (symptoms or behaviours), which are converted to five behavioural domains, with a supplementary three-item psychosis screen. It is administered as a semi-structured interview to the caregiver or family member about their observations of the pwALS and any associated behavioural impairment, changes, and/or symptoms. The five behavioural domains are: behavioural disinhibition (three items), apathy/inertia (one item), loss of sympathy/empathy (two items), perseverative/stereotyped/compulsive/ritualistic behaviour (two items), and hyperorality/altered eating behaviour (two items). The behavioural domain score can range from 0 (no behaviour impairment) to 5 (most behavioural impairment). Additionally, the behavioural score for symptoms or behaviours can range from 0 (no behaviour symptoms) to 10 (most behaviour symptoms). The three-item psychosis screen examines presence of hallucinations, bizarre beliefs/behaviours, and suspiciousness/persecutory feelings, ranging from 0 (no psychotic symptoms) to 3 (most psychotic symptoms). The interview is based on the guidelines and diagnostic criteria for behavioural variant FTD and other FTD conditions [38]. The ECAS is a recommended assessment for identifying behavioural and cognitive impairment categories (ALSbi, ALSci, ALScbi, ALSni), in line with the diagnostic criteria for ALS–FTSD [7].

#### QoL assessment

The ALSsQoL-Short Form (ALSsQoL-SF) [39] is a self-rated 20-item instrument that takes two to four minutes to complete, and assess ALS-specific subdomains of QoL. It was derived from the 50-item ALSsQoL Revised instrument using item response analysis [13]. There are a total of six subdomains examined: negative emotion (three items), physical symptoms (five items), bulbar function (two items), interaction with people and the environment (four items), religiosity (two items), and intimacy (four items). Each of the subdomain items are scored on an 11-point Likert scale ranging from 0 (strongly disagree) to 10 (strongly agree). Average item scores are used for the overall QoL score and for subdomain scores, with lower scores indicating worse QoL. All ALSsQoL-SF subdomains had an acceptable internal consistency reliability (Cronbach’s standardised alpha ≥ 0.70), as well as being validated against relevant psychosocial and physical functioning measures (e.g. McGill QoL Single-Item Scale, World Health Organization QoL assessment, Satisfaction with Life Scale, Center for Epidemiological Studies Depressed Mood Scale, 18-item Brief Symptom Inventory, Idler Index of Religiosity).

### Procedure

Data was routinely collected as a part of individuals’ standard of care visits to the Penn State Milton S. Hershey Medical Center’s Multidisciplinary ALS clinic, Hershey, Pennsylvania, USA. Clinical and demographic data (i.e. age, gender, date diagnosis, date symptom onset) were collected at the initial visit. Additionally, ALSFRS-R and QoL data are collected at regular intervals throughout the individual’s care. PwALS were seen every 3–6 months as a part of their standard care. The ECAS was administered by a trained clinical psychologist or speech and language pathologist, after the initial visit at a stage in their care deemed clinically relevant by the multidisciplinary care team. Data were entered into patient’s medical records and retrospectively collated, with all ALSFRS-R, QoL and ECAS assessment being completed within 3 months of the ECAS data.

This study has gained Pennsylvania State University Institutional Review Board ethical approval. Due to this being a retrospective analysis of secondary clinical data that was collected as a part of routine clinical care, additional consent from individuals was not required and consent waiver was in place.

### Statistical analysis

All data analysis was conducted using R statistical software [40]. The analysis looked to explore cognitive and behavioural impairment group differences on QoL and its subdomains. Following this, a more in-depth exploration of the association between specific impairments and different QoL domains was performed. The analysis plan is outlined below, with the threshold for statistical significance set at* p* < 0.05.

#### Data preparation

The dataset was examined for missingness and missing data were excluded from analysis. Individuals were categorised into cognitive and behavioural impairment group classifications (ALSbi, ALSci, ALScbi or ALSni) based on published cutoffs for the ECAS [35] in line with the ALS–FTSD diagnostic criteria [7]. ALSci was defined using the ECAS global score cutoff (≤ 105) and/or the ECAS ALS-specific score cutoff (≤ 77). ALSbi was defined as the presence of apathy or at least two non-overlapping behavioural impairment features. ALScbi was defined as being present when both ALSci and ALSbi criteria were met. The distribution and linearity of the data were assessed using Shapiro–Wilk tests of normality, in addition to visual inspection of histograms, which in turn determined whether use of parametric or non-parametric statistics was most appropriate.

#### Cross-sectional group comparison

Group comparison of descriptive and clinical data (i.e. age, gender, education, disease duration, ALSFRS-R score) were performed using univariate ANOVAs (with follow-up Tukey's honestly significant difference (HSD) tests for multiple comparisons). Kruskal–Wallis *H* test was used for group comparison (with follow-up post hoc Dunn tests) and Chi (*χ*^2^) squared tests for nominal or categorical variable group comparisons.

If the data were parametric and assumptions were met, group comparison was performed initially using a univariate analysis of variance (ANOVA) to explore differences between cognitive and behavioural impairment groups (ALSci, ALSbi, ALScbi, and ALSni) on the overall score for the ALSsQoL-SF instrument with follow-up post hoc Tukey's honestly significant difference (HSD) tests for multiple comparisons. If data were non-parametric, Kruskal–Wallis *H* test was used for group comparison with follow-up post hoc Dunn tests with a false discovery rate correction for multiple comparisons [41]. Furthermore, univariate ANOVAs with follow-up Tukey's HSD tests for multiple comparisons were used to further explore differences in cognitive and behavioural impairment groups across six domains (negative emotion, interaction with people and the environment, intimacy, physical functioning, bulbar function, religiosity) of the ALSsQoL-SF instrument. If data were non-parametric, Kruskal–Wallis *H* test was used for group comparison for each QoL subdomain with follow-up post hoc Dunn tests with a false discovery rate correction for multiple comparisons [41].

For parametric analysis (i.e. ANOVAs), *η*^2^ (eta squared) was used to quantify effect size [42] with values of up to 0.01 representing a small effect, 0.06 representing a medium effect and above 0.14 representing a large effect. Cohen’s *d* was used to quantify effect size for parametric post hoc tests [43] with values of up to 0.2 representing a small effect, around 0.5 representing a medium effect and above 0.8 representing a large effect. For non-parametric analysis (i.e. Kruskal–Wallis *H* test), *ε*^2^ (epsilon squared, a *eta*^2^ *H* based statistic) was used to compute effect size [44] with values of up to 0.01 representing a small effect, 0.06 representing a medium effect, and above 0.14 representing a large effect. Vargha and Delaney’s *A* was used to quantify effect size for non-parametric post hoc tests [45] with values of up to 0.56 representing a small effect, between 0.56 and 0.64 representing a medium effect, and above 0.71 representing a large effect.

#### Predictive analysis

Multiple hierarchical regression analysis (enter method) was performed to explore the predictive power of specific cognitive and behavioural predictors on overall scores for the ALSsQoL-SF and its subdomains as outcomes. The Step 1 model of the multiple hierarchical regression analysis involved entering baseline predictor variables of that were deemed to be influential towards QoL, such as age, gender, and any other variable emergent from the previous group analysis. The subsequent Step 2 model involved baseline predictor variables and addition of predictors of ECAS specific, nonspecific and behavioural (inclusive of psychosis) scores (referred to as overall cognitive and behavioural scores), with attention towards significant predictors, beta values, and percentage (%) variance explained by the model (*R*^*2*^). Further, ANOVA was used to compare if there was significant *R*^2^ change (△*R*^2^) between Step 1 model and Step 2 model.

Guided by significant overall cognitive and behavioural score predictor results from the Step 1 and Step 2 model comparison, a further Step 3 model was devised with specific ECAS Cognitive (verbal fluency, executive functioning, language, memory, visuospatial) and behavioural (behavioural disinhibition, apathy/inertia, loss of sympathy/empathy, perseverative/stereotyped/compulsive/ritualistic behaviour, and hyperorality/altered eating behaviour, inclusive of three psychosis subdomains) domains (referred to as specific cognitive and behavioural domain scores) as predictors of overall scores for the ALSsQoL-SF and its subdomains. Once again, particular attention was given towards significant predictors, beta values, and percentage (%) variance explained by the model (*R*^2^). ANOVA was used to compare if there was significant *R*^2^ change (△*R*^2^) between Step 1 model and Step 3 model on adjusted *R*^2^ change.

## Results

Out of 135 pwALS in the dataset, one had a diagnosis of comorbid FTD and was excluded from subsequent analysis. Of the remaining134, 9.7% (*N* = 13) had missing data relating to the main variables of interest (ALSFRS-R, ECAS, ALSsQoL-SF), which is less than 10% and acceptable for statistical inference. 3.7% (*N* = 5) of pwALS were excluded due to incomplete or missing ECAS data and 5.2% (*N* = 7) were excluded due to incomplete or missing ALSsQoL-SF data. Finally, one pwALS was excluded due to incomplete or missing both ALSsQoL-SF and ECAS data. This resulted in 121 pwALS being included in the analysis. Of the caregiver (spouses/partners) or family members who completed the ECAS behaviour interview, 62.8% (*N* = 76) were female.

### Clinical and demographic summary

Table 1 shows the overall clinic–demographic summary for the pwALS group, which has also been subdivided by the four King’s clinical disease stages. There was an overall significant difference between King’s stages on disease duration since onset of symptoms, ALSFRS-R and domain scores. Those in Stage 3 had significantly longer disease duration than those in Stage 2, with no other differences observed. Post hoc Dunn tests showed that those pwALS in Stage 4 had a significantly lower ALSFRS-R score than those in Stages 1 (*p* < 0.001) and Stage 2 (*p* < 0.001), with no significant difference compared to those in Stage 3 (*p* = 0.23). Those in Stage 3 had a significantly lower ALSFRS-R score than those in Stage 1 (*p* < 0.001) and Stage 2 (*p* = 0.003). Finally, those in Stage 2 had a significantly lower ALSFRS-R score than those in Stage 1 (*p* = 0.012). See Online Resource 1 for full reporting of ALSFRS-R subscale scores across King’s clinical disease stages.

**Table 1:** Clinico-demographic variables of pwALS in the dataset (*N* = 121), subdivided and compared by King's Clinical Disease Staging

	All (*N* = 121)	Kings Stage 1 (*N* = 22)	Kings Stage 2 (*N* = 35)	Kings Stage 3 (*N* = 34)	Kings Stage 4 (*N* = 30)	Statistic	*p* value
Age (mean, SD)	61.5 (9.9)	57.7 (11.1)	64.4 (9.5)	52.5 (9.8)	60.0 (8.6)	*H* = 6.53	0.09
Sex (M/F)	83/38	14/8	11/24	9/25	10/20	*χ*^2^ = 0.69	0.88
Handedness (L/R/A)	12/106/3	3/18/1	2/32/1	5/29/0	2/27/1	*χ*^2^ = 8.46	0.49
Education (mean, SD)	15.4 (2.7)^a^	15.4 (2.3)^b^	15.1 (2.9)^c^	15.6 (3.1)^d^	15.5 (2.4)^e^	*H* = 0.15	0.99
Site onset (bulbar/limb)	40/81	8/14	8/27	10/24	14/16	*χ*^2^ = 4.47	0.22
Disease duration (since onset), months (median, IQR)	21 (20)	18.5 (12.5)	18 (10)	27 (41.5)	27 (20.25)	*H* = 10.36	**0.015**
Disease duration (since diagnosis), months (median, IQR)	6 (9)	5 (6.75)	5 (4)	7 (15.75)	9 (12)	*H* = 7.03	0.07
ALSFRS-R (mean, SD) /48	34.0 (7.1)	40.7 (3.7)	34.7 (4.1)	31.7 (5.9)	28.6 (7.7)	*H* = 49.41	** < 0.001**
ECAS cognitive total % (*N*) impaired	46.3 (56)	40.9 (9)	48.6 (17)	52.9 (18)	40.0 (12)	*χ*^2^ = 1.41	0.70
ECAS cognitive (mean, SD)							
Language/28	25.5 (3.3)	24.3 (5.9)	25.9 (2.2)	25.4 (2.9)	25.9 (2.2)	*H* = 0.92	0.82
Verbal fluency/24	14.9 (5.6)	15.6 (5.6)	15.0 (4.8)	14.0 (6.9)	15.4 (4.9)	*H* = 0.68	0.89
Executive/48	37.2 (6.5)	36.5 (9.9)	37.0 (6.3)	36.7 (5.9)	38.4 (5.3)	*H* = 1.26	0.74
Specific/100	77.6 (12.4)	79.3 (19.4)	77.9 (9.9)	76.1 (11.9)	79.7 (8.7)	*H* = 1.48	0.69
Memory/24	16.6 (4.2)	15.7 (5.0)	16.0 (4.5)	16.6 (3.9)	17.8 (3.1)	*H* = 3.76	0.29
Visuospatial/12	11.5 (0.9)	11.1 (1.5)	11.7 (0.6)	11.5 (0.7)	11.7 (0.8)	*H* = 3.76	0.29
Nonspecific/36	28.1 (4.5)	26.8 (5.9)	27.7 (4.6)	28.1 (4.0)	29.5 (3.3)	*H* = 3.19	0.36
Total/136	105.7 (15.4)	103.1 (24.5)	105.6 (12.6)	104.2 (14.3)	109.2 (10.4)	*H* = 2.04	0.57
ECAS behaviour (median, IQR)							
Domains /5	0 (2)	0 (2)	0 (0.5)	0.5 (3)	0 (3)	*H* = 4.94	0.18
Psychosis /3	0 (0)	0 (0)	0 (0)	0 (0)	0 (0)	*H* = 1.92	0.59

Significant *p* values are in bold

*ALS* amyotrophic lateral sclerosis, *N* number, *M* male, *F* female, *L* left, *R* right, *A* ambidextrous, *SE* standard deviation, *IQR* interquartile range, *ALSni* ALS not impaired, *ALSci* ALS cognitive impairment, *ALSbi* ALS behavioural impairment, *ALScbi* ALS cognitive and behavioural impairment, *ALSFRS* ALS Functional Rating Scale Revised, *ECAS* Edinburgh Cognitive and Behavioural ALS Screen

^a^*N* = 80, ^b^*N* = 14, ^c^*N* = 23, ^d^*N* = 22, ^e^*N* = 21

There were no other significant differences observed on clinical or demographic variables. When exploring cognitive and behaviour impairment, there were no significant differences observed across the King’s clinical disease stages.

### Cognitive and behavioural impairment summary

Table 2 shows the comparisons of clinical, demographic, cognitive and behavioural scores across the ALS–FTSD diagnostic criteria classifications [7] based on ECAS scores.

**Table 2:** Clinico-demographic variables for pwALS in the dataset, subdivided and compared by Strong et al. [7] ALS–FTSD classifications (ALSci, ALSbi, ALScbi, ALSni)

	ALSni (*N* = 47)	ALSci (*N* = 35)	ALSbi (*N* = 17)	ALScbi (*N* = 22)	Statistic	*p* value
Age (mean, SD)	59.9 (9.6)	64.8 (8.4)	59.5 (11.4)	62.1 (9.9)	*H* = 5.46	0.14
Sex (M/F)	15/32	14/21	5/12	4/18	*χ*^2^ = 3.02	0.39
Handedness (L/R/A)	4/41/2	3/32/0	3/13/1	2/20/0	*χ*^2^ = 5.95	0.75
Education (mean, SD)	16.7 (2.3)^c^	13.5 (2.7)^d^	16.2 (2.5)^e^	13.8 (2.2)^f^	*H* = 21.80	** < 0.001**
Site onset (bulbar/limb)	19/28	12/23	4/13	4/18	*χ*^2^ = 2.94	0.40
Disease duration (since onset), months (median, IQR)	20 (14)	20 (12)	27 (22)	31 (23.75)	*H* = 7.06	0.07
Disease duration (since diagnosis), months (median, IQR)	6 (4)	5 (7.5)	10 (9)	7.5 (24.5)	*H* = 5.49	0.14
ALSFRS-R (mean, SD)/48	35.7 (5.7)	33.8 (8.1)	29.8 (7.0)	34.2 (7.2)	*H* = 8.50	**0.036**
Bulbar (mean, SD)	8.8 (3.1)	8.9 (3.1)	9.2 (2.8)	9.5 (2.3)	*H* = 0.73	0.73
Fine motor (mean, SD)	8.4 (3.1)	7.6 (3.5)	5.8 (4.0)	7.0 (3.6)	*H* = 5.56	0.14
Gross motor (mean, SD)	7.9 (3.3)	7.2 (3.3)	5.6 (2.6)	7.8 (2.8)	*H* = 7.99	**0.046**
Respiratory (mean, SD)	10.7 (1.8)	10.0 (3.0)	9.2 (2.5)	9.5 (2.8)	*H* = 7.35	0.06
ECAS cognitive total % (N) impaired	0 (0)	97.1 (34)^a^	0 (0)	100 (22)	*χ*^2^ = 117.10	** < 0.001**
ECAS cognitive (mean, SD)						
Language/28	27.0 (1.3)	24.4 (2.7)	26.8 (1.6)	22.6 (5.4)	*H* = 45.87	** < 0.001**
Verbal fluency/24	18.1 (2.9)	12.5 (5.1)	19.3 (2.4)	8.4 (4.8)	*H* = 57.95	** < 0.001**
Executive/48	41.4 (3.2)	33.1 (5.6)	39.6 (4.2)	32.3 (8.0)	*H* = 55.70	** < 0.001**
Specific/100	86.6 (4.4)	70.0 (7.6)	85.5 (4.8)	63.3 (14.0)	*H* = 84.64	** < 0.001**
Memory/24	18.6 (2.3)	14.8 (4.1)	18.7 (2.4)	13.2 (5.0)	*H* = 40.26	** < 0.001**
Visuospatial/12	11.6 (0.9)	11.7 (0.7)	11.6 (0.7)	11.0 (1.3)	*H* = 7.12	0.07
Nonspecific/36	30.2 (2.6)	26.5 (4.2)	30.4 (2.5)	24.2 (5.7)	*H* = 37.71	** < 0.001**
Total/136	116.8 (5.0)	96.5 (7.9)	116.0 (5.3)	87.5 (18.7)	*H* = 90.23	** < 0.001**
ECAS behaviour (median, IQR)						
Domains/5	0 (0)	0 (0)	3 (2)	5 (2)	*H* = 100.11	** < 0.001**
Symptoms/10	0 (0)	0 (0)	3 (2)	5.5 (5)	*H* = 99.73	** < 0.001**
Psychosis/3	0 (0)	0 (0)	0 (0)	0 (0)	*H* = 20.25	** < 0.001**
ECAS behaviour domains % (*N*)						
Only apathy or inertia present	0 (0)	0 (0)	17.6 (3)	0 (0)	*χ*^2^ = 2.09	0.15^b^
2 non-overlapping behavioural domains present	0 (0)	0 (0)	82.4 (14)	100 (22)		

*ALS* amyotrophic lateral sclerosis, *N* number, *M* male, *F* female, *L* left, *R* right, *A* ambidextrous, *SE* standard Deviation, *IQR* interquartile range, *ALSni* ALS not impaired, *ALSci* ALS cognitive impairment, *ALSbi* ALS behavioural impairment, *ALScbi* ALS cognitive and behavioural impairment, *ALSFRS* ALS Functional Rating Scale Revised, *ECAS* Edinburgh Cognitive and Behavioural ALS Screen

^a^*N* = 1 was not impaired on the ECAS Cognitive total, but showed impairment on the ECAS ALS-specific score

^b^Statistical comparison only between ALSbi and ALScbi. Significant *p* values are in bold

^c^*N* = 35, ^d^*N* = 16, ^e^*N* = 12, ^f^*N* = 17

When applying these diagnostic criteria classifications, 61.2% (*N* = 74) had either cognitive and/or behavioural impairment. 28.9% (*N* = 35) were classified as ALSci, 14.1% (*N* = 17) as ALSbi, and finally 18.2% (*N* = 22) as ALScbi. 38.8% (*N* = 47) were classified as ALSni.

The frequencies of specific behavioural impairments were 22.3% (*N* = 27) for behavioural disinhibition, 25.6% (*N* = 31) for apathy/inertia, 25.6% (*N* = 31) for loss of sympathy/empathy, 26.4% (*N* = 32) for perseverative/stereotyped/compulsive/ritualistic behaviour, and 20.7% (*N* = 25) for hyperorality/altered eating behaviour. 7.4% (*N* = 9) showed at least one element of psychosis, with 5.8% (*N* = 7) experiencing suspiciousness/persecutory feelings, 5.0% (*N* = 6) experiencing bizarre beliefs/behaviours, and only 0.8% (*N* = 1) experiencing hallucinations.

There was a significant difference in years of education across ALS–FTSD diagnostic criteria classifications. Post hoc Dunn tests showed that individuals with ALSci had significantly fewer years of education than those with ALSbi (*p* = 0.016) and ALSni (*p* < 0.001). Additionally, those with ALScbi also had significantly less years of education than those with ALSbi (*p* = 0.030) and ALSni (*p* = 0.002). There were no other differences on years of education.

A further significant difference was observed on the ALSFRS-R and the gross motor domain across ALS–FTSD classifications. Post hoc test showed that for the overall ALSFRS-R, only ALSbi had a significantly lower score than ALSni (*p* = 0.022), which was specifically observed on the gross motor domain (*p* = 0.036). No other differences were observed on the ALSFRS-R.

A comparison of ECAS scores across ALS–FTSD diagnostic criteria classifications showed a pattern reflective of each cognitive and behavioural subdomain. Post hoc test showed that those with ALSci had significantly worse cognitive impairment (or lower ECAS scores) compared to those with ALSbi (ECAS Cognitive Nonspecific *p* = 0.008; Cognitive Specific *p* < 0.001; Total *p* < 0.001) and ALSni (ECAS Cognitive Nonspecific *p* = 0.008; Cognitive Specific *p* < 0.001; Total *p* < 0.001) categories, with no significant difference compared to those with ALScbi. No difference was observed between ALSbi and ALSni on cognitive scores (ECAS Cognitive Nonspecific, Cognitive Specific or Total scores). In terms of behaviour, ALSbi has significantly higher ECAS behavioural interview domain and symptom scores compared to ALSni (*p* < 0.001) and ALSci (*p* < 0.001), but no significant difference compared to those with ALScbi. Individuals with ALScbi had significantly worse cognitive impairment or lower ECAS scores (ECAS cognitive nonspecific *p* < 0.001; cognitive specific *p* < 0.001; Total *p* < 0.001) and higher behavioural impairment scores (domain and symptom scores *p*s < 0.001) than those with ALSni. While there was an overall between-group difference in experiences with psychosis, this appeared to be driven by presence in those with ALSbi (23.5%, *N* = 4) and ALScbi (22.7%, *N* = 5), compared to ALSci and ALSni in whom no psychosis was observed.

### Cognitive/behavioural impairment-QoL group comparison

There was a significant difference across ALS–FTSD diagnostic criteria classifications (ALSni, ALSci, ALSbi, ALScbi) on the overall ALSsQoL-SF score (*F*(3, 117) = 4.14, *p* = 0.008, *η*^2^ = 0.10). Tukey's HSD post hoc tests showed that those in the ALSbi group (*M* = 6.0, SD = 1.2) had overall lower QoL than those with ALSci (*M* = 7.3, SD = 1.3) group (*p* = 0.005, *d* = 0.99), with no other group differences observed.

**Fig. 1:**
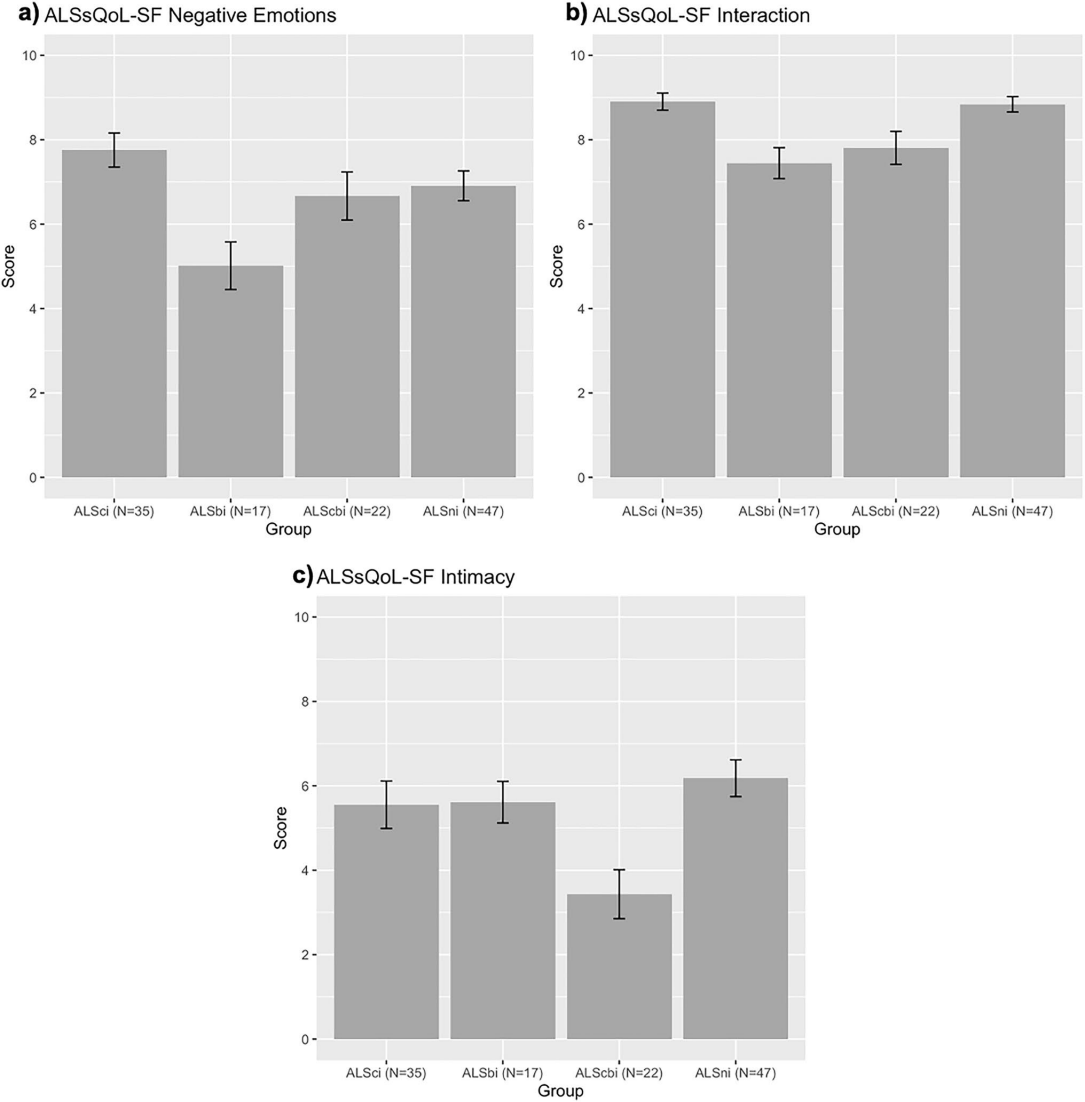
Comparison between ALSci, ALSbi, ALScbi, ALSni groups on ALSsQoL-SF subdomain scores: a) negative emotions b) interaction with people and the environment c) intimacy. Lower scores indicate worse QoL

Figure 1 shows comparison of significant ALSsQoL-SF subdomains based on ALS–FTSD diagnostic criteria classifications, demonstrating a significant difference between cognitive and behaviour impairment groups on three of the five different subdomains of QoL: negative emotions (*H*(3) = 13.15, *p* = 0.004, *ε*^2^ = 0.09), interaction with people and the environment (*H*(3) = 16.92, *p* < 0.001,* ε*^2^ = 0.12), and intimacy (*H*(3) = 11.57, *p* = 0.009, *ε*^2^ = 0.07).

For negative emotions (see Fig. 1a), post hoc tests showed the ALSbi group (*M* = 5.0, SD = 2.3) had significantly worse QoL relating to this domain when compared with the ALSci (*M* = 7.8, SD = 2.4) group (*p* = 0.002, *A* = 0.79) and ALSni (*M* = 6.9, SD = 2.4) group (*p* = 0.04, *A* = 0.73), with no other differences observed. For interaction with people and the environment (see Fig. 1b), post hoc test showed that those once again in the ALSbi group (*M* = 7.5, SD = 1.5) had significantly worse QoL related with this domain compared to those in the ALSci (*M* = 8.9, SD = 1.2) group (*p* = 0.003, *A* = 0.79) and ALSni (*M* = 8.8, SD = 1.3) group (*p* = 0.002,* A* = 0.80). Furthermore, those in the ALScbi group (*M* = 7.8, SD = 1.8) had significantly lower QoL related to interaction with people and the environment compared to the ALSci (*M* = 8.9, SD = 1.2) group (*p* = 0.044, *A* = 0.67), with trending to significant differences when compared to the ALSni (*M* = 8.8, SD = 1.3) group (*p* = 0.053, *A* = 0.65). No other differences were observed on the QoL relating to interaction with people and the environment subdomain. When exploring intimacy (see Fig. 1c), post hoc tests showed that the ALScbi group (*M* = 3.4, SD = 2.7) had lower QoL in this domain than those in the ALSni (*M* = 6.2, SD = 3.0) group (*p* = 0.005, *A* = 0.74) and also the ALSci (*M* = 5.6, SD = 3.3) group (*p* = 0.033, *A* = 0.70), with no other differences observed. See Online Resource 2 for full table comparison of QoL subdomains across ALS–FTSD classifications.

### Cognitive/behavioural predictors of QoL

For the overall ALSsQoL-SF score (see Table 3), the Step 1 model explained 4.4% of the variance, with only ALSFRS-R shown as a significant positive predictor (*β* = 0.20, *p* = 0.03) and no such relationship between age or gender. In the Step 2 model, following entry of overall cognitive and behavioural (inclusive of psychosis) scores, only psychosis was a significant negative predictor of the overall ALSsQoL-SF score (*β*  = − 0.21, *p* = 0.04). There was no significant *R*^2^ change between the Step 1 and Step 2 models (*F*(4, 113) = 2.00, *p* = 0.100). Furthermore, none of the specific psychosis domain scores significantly predicted the overall ALSsQoL-SF score, with no significant difference between the % variance accounted for between the Step 1 and Step 3 models (*F*(3, 114) = 2.04, *p* = 0.112).

**Table 3:** Hierarchical stepwise multiple regression results with ALSsQoL-SF overall score as an outcome

	*b*	*β* [95% CI]	*p*	*R*^2^
Step 1				0.044
Age	0.01	0.04 [0.02, 0.06]	0.66	
Gender	− 0.06	− 0.02 [− 0.52, 0.48]	0.82	
ALSFRS-R	**0.04**	**0.20 [0.17, 0.24]**	**0.03**	
Step 2				0.108
Age	0.001	0.01 [− 0.02, 0.03]	0.94	
Gender	− 0.04	− 0.01 [− 0.51, 0.49]	0.88	
ALSFRS-R	0.03	0.15 [0.11, 0.18]	0.12	
Specific cognitive	− 0.02	− 0.20 [− 0.21, − 0.17]	0.10	
Nonspecific cognitive	0.02	0.06 [− 0.002, 0.13]	0.58	
Behaviour	− 0.06	− 0.08 [− 0.23, 0.06]	0.43	
Psychosis	**− 0.61**	**− 0.21 [− 0.79, 0.37]**	**0.04**	
Step 3				0.093
Age	0.004	0.02 [0.004, 0.05]	0.76	
Gender	− 0.04	− 0.01 [− 0.51, 0.49]	0.88	
ALSFRS-R	0.03	0.16 [0.12, 0.19]	0.10	
Bizarre beliefs/behaviours	− 0.00	− 0.001 [− 1.37, 1.37]	1.00	
Hallucinations	− 1.79	− 0.12 [− 2.95, 2.70]	0.21	
Suspiciousness/persecutory feelings	− 0.83	− 0.15 [− 1.44, 1.14]	0.21	

Significant *p* values are in bold

*b*  unstandardised beta, *β* standardised beta, *CI* confidence interval, *ALSFRS-R* ALS Functional Rating Scale Revised

For ALSsQoL-SF subdomain scores, there was significance relating to cognitive and behavioural domain predictors. For the interaction with people and the environment subdomain (see Table 4), while the Step 1 model explained 2.4% of the variance with no significant age, gender, and ALSFRS-R predictors, the Step 2 model showed the overall psychosis score as a significant negative predictor of this QoL subdomain (*β*  = − 0.32, *p* = 0.001), with the behaviour score trending towards significance (*β*  = − 0.18, *p* = 0.06). Supplementarily, the Step 2 model accounted for significantly more variance (△*R*^2^ = 0.159) compared to the Step 1 model (*F*(4, 113) = 2.04, *p* < 0.001). Further, in exploring specific behavioural and psychosis domain scores through the Step 3 model, the loss of sympathy/empathy domain (*β*  = − 0.28, *p* = 0.03), suspiciousness/persecutory feelings (*β*  = − 0.30, *p* = 0.007), and hallucinations (*β*  = − 0.21, *p* = 0.03) scores were significant negative predictors for QoL associated with interaction with people and the environment. However, it is important to note that only one individual exhibited hallucinations. The Step 3 model also accounted for significantly more variance (△*R*^2^ = 0.246) compared to the Step 1 model (*F*(8, 109) = 4.59, *p* < 0.001).

**Table 4:** Hierarchical stepwise multiple regression results with ALSsQoL-SF interaction with people and the environment subdomain score as an outcome

	*b*	*β* [95% CI]	*p*	*R*^2^
Step 1				0.024
Age	0.01	0.08 [0.05, 0.11]	0.40	
Gender	− 0.30	− 0.10 [− 0.68, 0.49]	0.30	
ALSFRS-R	0.02	0.09 [0.05, 0.12]	0.36	
Step 2				0.183
Age	0.01	0.04 [0.01, 0.06]	0.67	
Gender	− 0.22	− 0.07 [− 0.61, 0.05]	0.43	
ALSFRS-R	− 0.002	− 0.01 [− 0.05, 0.02]	0.89	
Specific	− 0.02	− 0.13 [− 0.15, − 0.10]	0.24	
Non-specific	0.01	0.04 [− 0.03, 0.11]	0.68	
Behaviour	− 0.15	− 0.18 [− 0.35, − 0.02]	0.06	
Psychosis	**− 1.07**	**− 0.32 [− 0.96, 0.31]**	**0.001**	
Step 3				0.270
Age	0.01	0.05 [0.03, 0.08]	0.54	
Gender	− 0.15	− 0.05 [− 0.59, 0.50]	0.59	
ALSFRS-R	− 0.005	− 0.02 [− 0.06, 0.01]	0.79	
Behavioural disinhibition	− 0.21	− 0.06 [− 1.06, 0.95]	0.68	
Apathy/inertia	− 0.78	− 0.22 [− 1.14, 0.68]	0.09	
Loss of sympathy/empathy	**− 0.97**	**− 0.28 [− 1.17, 0.60]**	**0.03**	
Perseverative/stereotyped/compulsive/ritualistic behaviour	0.55	0.16 [− 0.66, 0.99]	0.19	
Hyperorality/altered eating behaviour	0.72	0.20 [− 0.71, 1.10]	0.12	
Bizarre beliefs/behaviours	0.48	0.07 [− 1.46, 1.60]	0.53	
Hallucinations	**− 3.43**	**− 0.21 [− 3.31, 2.90]**	**0.03**	
Suspiciousness/persecutory feelings	**− 1.91**	**− 0.30 [− 1.69, 1.09]**	**0.007**	

Significant *p* values are in bold. *b* unstandardised beta, *β* standardised beta, *CI* confidence interval, *ALSFRS-R* ALS Functional Rating Scale Revised

For the negative emotions subdomain (see Table 5), the Step 1 model explained 2.9% of the variance, with no significant age, gender or ALSFRS-R predictors. The Step 2 model showed there were two significant negative predictors of negative emotions subdomain of the ALSsQoL-SF, specifically the ALS-specific score (*β*  = − 0.23, *p* = 0.04) and psychosis score (*β*  = − 0.28, *p* = 0.007). This was supported by a significant increase in variance accounted of the Step 2 model (△*R*^2^ = 0.109) when compared to the Step 1 model (*F*(4, 113) = 3.58, *p* = 0.009). Specific cognitive and psychosis domain score exploration through the Step 3 model showed that the verbal fluency score (*β*  = − 0.20, *p* = 0.047) and suspiciousness/persecutory feelings score (*β*  = − 0.28, *p* = 0.01) were both significant negative predictor for QoL associated with negative emotions. There was a further difference with the Step 3 model accounting for significantly higher variance (△*R*^2^ = 0.121) than the Step 1 model (*F*(6, 111) = 2.65, *p* = 0.02).

**Table 5:** Hierarchical stepwise multiple regression results with ALSsQoL-SF negative emotions subdomain score as an outcome

	*b*	*β* [95% CI]	*p*	*R*^2^
Step 1				0.029
Age	0.01	0.05 [0.005, 0.10]	0.57	
Gender	0.65	0.12 [− 0.88, 1.11]	0.20	
ALSFRS-R	0.04	0.12[0.05, 0.18]	0.20	
Step 2				0.138
Age	− 0.0001	− 0.0001 [− 0.05, 0.05]	1.00	
Gender	0.69	0.13 [− 0.84, 1.09]	0.16	
ALSFRS-R	0.01	0.03 [− 0.03, 0.10]	0.73	
Specific	**− 0.05**	**− 0.23 [− 0.28, − 0.18]**	**0.04**	
Non-specific	0.0001	0.0001 [− 0.12, 0.12]	1.00	
Behaviour	− 0.18	− 0.12 [− 0.41, 0.16]	0.22	
Psychosis	**− 1.56**	**− 0.28 [− 1.40, 0.85]**	**0.007**	
Step 3				0.150
Age	0.002	0.01 [− 0.04, 0.06]	0.91	
Gender	0.60	0.11 [− 0.85, 1.07]	0.22	
ALSFRS-R	0.02	0.05 [− 0.02, 0.11]	0.63	
Language	− 0.03	− 0.04 [− 0.22, 0.14]	0.75	
Verbal fluency	**− 0.09**	**− 0.20 [− 0.29, − 0.11]**	**0.047**	
Executive	0.00	0.004 [− 0.08, 0.09]	0.97	
Bizarre beliefs/behaviours	− 0.60	− 0.05 [− 3.09, 2.99]	0.70	
Hallucinations	− 0.48	− 0.02 [− 5.70, 7.66]	0.87	
Suspiciousness/persecutory feelings	**− 3.07**	**− 0.28 [− 2.77, 2.21]**	**0.01**	

Significant *p* values are in bold

*b* unstandardised beta, *β* standardised beta, *CI* confidence interval, *ALSFRS-R* ALS Functional Rating Scale Revised

In terms of intimacy (see Table 6), the Step 1 model explained 3.0% of the variance with no significant predictors relating to age, gender, and ALSFRS-R. The Step 2 model showed that only the overall behavioural score was a significant negative predictor of the intimacy QoL subdomain score (*β*  = − 0.22, *p* = 0.03) and accounted for significantly more variance (11.0%) change when compared to the Step 1 model (*F*(4, 113) = 2.53, *p* = 0.044). Further exploration of specific behavioural domains scores found that perseverative/stereotyped/compulsive/ritualistic behaviour domain was the only significant predictor of intimacy-related QoL (*β*  = − 0.30, *p* = 0.03); however, there was no significant change in variance accounted for when compared to the Step 1 model (*F*(8, 109) = 1.67, *p* = 0.11).

**Table 6:** Hierarchical stepwise multiple regression results with ALSsQoL-SF intimacy subdomain score as an outcome

	*b*	*β* [95% CI]	*p*	*R*^2^
Step 1				0.030
Age	− 0.05	− 0.15 [− 0.21, − 0.09]	0.11	
Gender	− 0.63	− 0.10 [− 1.28, 1.09]	0.30	
ALSFRS-R	− 0.003	− 0.007 [− 0.09, 0.07]	0.94	
Step 2				0.110
Age	− 0.04	− 0.14 [− 0.20, − 0.09]	0.12	
Gender	− 0.41	− 0.06 [− 1.23, 1.10]	0.49	
ALSFRS-R	− 0.01	− 0.01 [− 0.09, 0.07]	0.89	
Specific	0.01	0.04 [− 0.02, 0.10]	0.72	
Non-specific	0.09	0.14 [− 0.01, 0.29]	0.22	
Behaviour	**− 0.37**	**− 0.22 [− 0.56, 0.12]**	**0.03**	
Psychosis	0.43	0.06 [− 1.30, 1.42]	0.53	
Step 3				0.136
Age	**− 0.06**	**− 0.19 [− 0.24, − 0.13]**	**0.047**	
Gender	0.46	0.07 [− 1.28, 1.14]	0.45	
ALSFRS-R	− 0.03	− 0.06 [− 0.14, 0.02]	0.51	
Behavioural disinhibition	− 0.24	− 0.03 [− 2.27, 2.20]	0.83	
Apathy/inertia	− 0.69	− 0.10 [− 2.11, 1.91]	0.50	
Loss of sympathy/empathy	− 0.51	− 0.07 [− 2.05, 1.90]	0.61	
Perseverative/stereotyped/compulsive/ritualistic behaviour	**− 2.06**	**− 0.30 [− 2.14, 1.54]**	**0.03**	
Hyperorality/altered eating behaviour	1.42	0.19 [− 1.83, 2.21]	0.17	
Bizarre beliefs/behaviours	0.14	0.01 [− 3.39, 3.41]	0.94	
Hallucinations	− 2.89	− 0.09 [− 6.99, 6.81]	0.41	
Suspiciousness/persecutory feelings	1.06	0.08 [− 3.00, 3.16]	0.50	

Significant *p* values are in bold

*b* unstandardised beta, *β* standardised beta, *CI* confidence interval, *ALSFRS-R* ALS Functional Rating Scale Revised

In terms of bulbar function, the ALS nonspecific score was found to be a significant positive predictor (*β*  = 0.22, *p* = 0.045) for this QoL subdomain, as well as the ALSFRS-R score (*β*  = 0.22, *p* = 0.02), but there was no significant difference in the adjusted *R*^2^ change with the Step 1 model which included age, gender, and ALSFRS-R score (*F*(4, 113) = 1.46, *p* = 0.22) and no specific cognitive subdomain that was a significant predictor. Both overall cognitive and behavioural scores as well as the specific domain scores were found not to be significant predictors of remaining ALSsQoL-SF subdomains of religiosity and physical function. See Online Resource 3 for full reporting of QoL related to bulbar function, physical function, and religiosity analyses.

## Discussion

This study shows that cognitive and behavioural impairments have a negative effect on self-perceived QoL for pwALS, specifically in relation to certain disease-specific subdomains of QoL. These findings seem to be indicative of behavioural impairments being predictive of worse QoL, particularly relating to negative emotions or mental health and interaction with other as well as the environment. These findings may further suggest that behavioural impairment in combination with cognitive impairment may have negative bearing on intimacy and QoL associated with this.

Behavioural impairment has been frequently observed and linked to increased caregiver burden or strain in ALS [46] showcasing the external and environmental negative effects of this type of impairment. However, this study suggests that the external and environmental influence of this type of impairment might also be recognised by the pwALS and might be expressed through self-perceived QoL. This is relevant to previous research suggesting that deficits in insight and awareness (or anosognosia) towards behavioural impairment might be present in ALS [47, 48], a finding observed and documented in FTD, including ALS–FTD [3, 49, 50]. While some pwALS may not be aware of their own behavioural impairments, there might be secondary impact of these impairments on their QoL. This might then be internalised as negative effects on mental health, which is then expressed as worse QoL in the negative emotions subdomain by the pwALS. Further, this can also account for findings that feeling of strain or distress experienced by caregivers and family members relate to behavioural impairments; they observed that pwALS need further support relating to behavioural impairments that might not be related to physical or functional deterioration. In terms of the findings of this study, self-perceived QoL relating to interaction with people and the environment was found to be worse for pwALS, which may be a reciprocal expression of caregiver burden or strain relating to behavioural impairment. Importantly, there is a lack of disease specific, valid, and reliable self-perceived (self-reported or self-rated) behavioural impairment measures for use in ALS [51, 52], which may highlight a juxtaposition of comparing caregiver-rated behavioural impairment and self-perceived QoL. Further research would benefit from exploring self-perceived behavioural impairments, but also deficits of insight and awareness (or anosognosia) for these impairments, and their effect on or relationship with the QoL not only of the pwALS, but also of the caregivers and family members.

Specific cognitive and behavioural impairments were found to be predictive of certain QoL subdomains in ALS. In terms of behavioural impairment, loss of sympathy and empathy was found to be a negative predictor of QoL associated with interaction with people and the environment. Lower QoL in this subdomain has been characterised by lack of enjoyment of their surroundings, less support, unresponsiveness, and unsatisfying relationships for pwALS [14, 39]. Loss of sympathy and empathy often is characterised by decreased responsiveness to the needs and feelings of others, and by social disinterest, lack of interrelatedness, and less personal warmth. This can result from difficulty with social cognition, which has been identified as increasingly relevant in making inferences about the mental states of others, regulation of emotions or feelings, and emotional or empathic expressivity [53]. Further, loss of sympathy and empathy may also conceptually overlap with emotional apathy (defined as an indifference and emotional/affective neutrality, blunting, or flatness), which has been observed in bvFTD and other dementias [54]. Emotional apathy and social cognition difficulties seem to run parallel to the mentioned characteristics of the interaction with people and the environment QoL subdomain; however more in-depth neuropsychiatric or neuropsychological assessment may be necessary to elucidate specific QoL links. Again, while there may be a lack of awareness or insight for the behavioural impairments or symptoms themselves by the pwALS, it may be that these behavioural impairments are self-perceived by the pwALS through the aforementioned QoL subdomain. This may further tie in with the dynamic of caregiver burden or strain impacting the pwALS and also a shifting of services supporting caregivers or family members when more severe impairments or dementia is present.

Verbal fluency as a measure of cognitive initiation was found to be a negative predictor of QoL subdomain associated with negative emotions, characterised by feeling depressed, hopeless, and sad. This was the only QoL finding relating to a specific cognitive impairment, which may partly be due to the verbal fluency deficit begin the most common impairment in ALS [55, 56]. As such, this may potentially have practical everyday impact for pwALS. Problems with cognitive initiation may negatively impact the expression of thoughts or emotional states, which may result in further internalisation of these types of problems. Expression of this internalisation may manifest as dissatisfaction with emotional well-being and also negative emotions, with a lower psychological QoL in this domain. Previous research in other conditions has found an association between depression and verbal fluency deficits; however, more as a marker of general cognitive impairment observed in healthy or normative populations [57]. Relevant to this, the verbal fluency deficit has been shown to associate with characteristic initiation apathy subtypes in ALS [58], which may mediate or influence differential impacts on negative emotion-related QoL; however, further research would need to explore this. This may underscore the importance of support and provision to help externalise negative emotions for pwALS, as not doing so may have a negative effect on QoL. Psychotherapeutic approaches may therefore be helpful alongside considered scaffolding, functional and neuropsychological rehabilitation techniques that involve pwALS and their families.

Furthermore, there was a finding of lower QoL in the intimacy subdomain relating to a combination of cognitive and behavioural impairment for pwALS. One previous study found that lower caregiver-perceived marital intimacy was predicted by cognitive and behavioural impairment [59]. Previous research in bvFTD has shown that there is an overall decrease in the level and initiation of affection in addition to lower reciprocal intimate behaviour, with only some people with bvFTD displaying what can be classed as aberrant or unusual sexual behaviours [60]. Conversely, one small scale pilot study showed that there was a difference in terms of obsessiveness and aggressiveness in the context of intimacy for pwALS, from the perspectives of the caregivers, [61] with a further single case study showcasing inappropriate sexually behaviour in a case of ALS–FTD [62]. Notably, this current study is the first to showcase self-perceived QoL for intimacy being lower relative to cognitive and behavioural impairment, which might be specifically localised to perseverative/stereotyped/compulsive/ritualistic behaviours. These types of behaviours when observed in bvFTD are often characterised through repetitive and inflexible routines, rigidity in behaviour or thought, and obsessive as well as compulsive behaviours, wherein deviation or changes from these can cause significant distress for the individual. Speculatively, in terms of physical and emotional intimacy, these types of impairments can be a barrier for both the pwALS and also the partner or spouse (who is likely the caregiver), where there might be a decrease in communication about intimacy due to these impairments, leading to less initiation and reciprocal action in terms of intimacy. However, as noted in a recent large-scale systematic review [28] intimacy is an important area of QoL but is under-researched in ALS, particularly within the context of cognitive and behavioural impairment or associated FTD.

Psychosis was observed to be important in its association with specific QoL subdomains, particularly for negative emotions and interaction with people and the environment. While this study is in line with previous research showing psychosis occurs in approximately 10% of pwALS [9, 63, 64], it is also the first to suggests that it can negatively affect QoL. Previous research in bvFTD and those with ALS–FTD has found perceptual and psychosis-like disturbances to occur more prevalently in carriers of the *C9ORF72* gene [65], with these individuals having cognitive and behavioural impairment [66, 67]. While this has not been explored specifically with pwALS, a multitude of negative impacts have been observed relating to functional activities, wellbeing and QoL for people experiencing both their first episode of psychosis [68] and living with psychosis-related conditions [69]. In particular, this current study showed that suspiciousness/persecutory feelings (akin to paranoia) were found to be key negative predictors of these QoL internal (negative emotions) and external (interaction with people and the environment) subdomains for pwALS. This type of paranoid ideation, in terms of feeling persecuted or judged by others, has been shown to occur for pwALS, along with other psychotic experiences [70]. It is understandable that these paranoid types of thoughts and feelings can impact an individuals’ psychosocial QoL. This might relate to internal factors of depression as well as external factors in terms of feeling unsupported, feeling threatened, having less pleasure in (or towards) their surroundings, and having unsatisfying relationships. Mechanistically feelings of paranoia related to poorer QoL may become a vicious perpetuating cycle in the form of internalising negative emotions and externally perceiving that others and their environment are not helping or are a threat, which may have an effect on caregivers or family members as well as potentially on healthcare professionals. This may provide a further potential for application of psychotherapeutic approaches, therapies or strategies that work with pwALS and caregiver or family members to foster understanding of the cognitive and behavioural sequela of the condition. However, as psychosis is a complex and multifaceted phenomenon [71, 72] it requires further in-depth exploration, specifically in pwALS, to determine the interplay of QoL, psychosis and other neuropsychiatric symptoms.

This study has limitations. Although the data used was initially collected primarily to guide clinical care, it may not be representative of pwALS in terms of cognitive or behavioural impairment, as this may have only been assessed when deemed clinically relevant. Further to this, as QoL and ALSFRS-R data were collated within 3 months of the ECAS was administered, the results may have been affected by the temporal stability of the measures used and them not being collected at the same time. While the data collected was culturally based in the USA, UK cutoffs for the ECAS Cognitive element were utilised because they have been validated against a gold-standard neuropsychological battery [37]. A recent study proposed USA-specific ECAS Cognitive cutoffs [73]; however these were derived through statistical methodology and have not been extensively validated. However, the prevalence of cognitive and behavioural impairment in this dataset was 61.2%, which was within the range of previous research [9]. While the pwALS were well characterised clinically and demographically, certain elements such as trained clinician derived King’s stages (rather ALSFRS-R derived staging) and genetic status were not available in terms of common variants (e.g. *C9ORF72*). Further in-depth reliable clinical staging and understanding of the genotype–phenotype link with cognitive and behavioural impairment would be useful in future studies, particularly with reference to QoL. Additionally, there were limitations in the dataset due to missingness for years of education and socioeconomic status and no available data on ethnicity. Due to the convenience sampling nature of this study, there may be sample size limitations that may have impacted adequacy of statistical power, particularly as certain ALS–FTSD diagnostic criteria classifications groups were less common than others. However, the prevalence, frequency and characteristics of impairments are broadly in line with previous research [9, 11, 63]. There was also limited information available on the background, demographics and health of caregivers or family members of pwALS. This was due to the clinical, medical record nature of the dataset containing only relevant data to pwALS, which restricted further exploration of caregiver variables relative to self-perceived QoL of pwALS.

Future research should look to explore the relationship between caregiver strain or burden and self-perceived QoL for pwALS, with cognitive and behavioural impairment as a mediator. This may help promote understanding of the shared influence of these in the family dynamic or system, as the disease progresses and across disease stages. Finally, further investigation of longitudinal trajectories relating to QoL and its subdomains for pwALS, associated with cognitive and behavioural impairment, would be useful to understand the corresponding evolution of these constructs throughout the disease process. From a practical standpoint, the fast-progressing nature of ALS supplemented by potential development of cognitive or behavioural impairment may make it difficult for deployment of supportive approaches for QoL of pwALS in a timely manner. As such, the fast-progression of ALS may make QoL-associated interventions rapidly redundant, as priorities for the pwALS may be fast changing alongside disease progression. While there has been a recent advance in psychological therapy interventions for pwALS, specifically acceptance and commitment therapy [74], the moderating effect or interplay between cognitive or behavioural impairment and QoL is not clear. As such there may be a requirement for adaptations, as well as supportive or systemic approaches, to facilitate psychological interventions relative to these impairments. Future research would benefit from determining the utility of regular monitoring of QoL, and of cognitive or behavioural impairment to assess whether this would be helpful for well-timed and appropriate interventions.

In conclusion, behavioural impairments (inclusive of psychosis) have a relationship with self-perceived QoL for pwALS, specifically relating to externalisation towards environment and people around the individual but also in terms of internalisation associated with mental health difficulties. Additionally, both cognitive and behavioural impairment are significant factors for QoL relating to intimacy for pwALS. Overall this emphasises the importance of disease-specific monitoring and assessment of self-perceived QoL in the context of cognitive and behavioural impairments, as they may impact the pwALS implicitly. This has implications for further research to elucidate this complex topic and furthermore for clinical practice in terms of psychoeducation, management, and support for QoL for pwALS and their families who are experiencing the impacts of cognitive and/or behavioural impairment.

## Supplementary Information

Below is the link to the electronic supplementary material.

Supplementary file1 (DOCX 14 KB)

Supplementary file2 (DOCX 15 KB)

Supplementary file3 (DOCX 18 KB)
