# Supplementary material for: Self-perceived quality of life, cognitive and behavioural impairment in amyotrophic lateral sclerosis
Source: J Neurol. 2024 Aug 28;271(10):6822–38. doi: 10.1007/s00415-024-12639-z (PMC11446980; doi:10.1007/s00415-024-12639-z)
Supplement: Supplementary file 1 — Supplementary file1 (DOCX 14 KB) [file 415_2024_12639_MOESM1_ESM.docx]

Online Resource 1.

ALSFRS-R subscores across King’s Clinical Disease Stages

|  | **All**  **(N= 121)** | **Kings Stage 1**  **(N= 22)** | **Kings Stage 2**  **(N= 35)** | **Kings Stage 3**  **(N= 34)** | **Kings Stage 4**  **(N= 30)** | **Statistic** | ***p* value** |
| --- | --- | --- | --- | --- | --- | --- | --- |
| Bulbar (mean, SD) | 9.0 (3.1) | 9.8 (2.9) | 10.6 (2.5) | 8.8 (2.2) | 7.1 (3.6) | H = 23.76 | **<0.001** |
| Fine Motor (mean, SD) | 7.6 (3.5) | 10.0 (2.9) | 7.8 (2.8) | 5.9 (3.4) | 7.3 (3.9) | H = 22.36 | **<0.001** |
| Gross Motor (mean, SD) | 7.3 (3.2) | 9.6 (3.1) | 7.1 (2.7) | 6.3 (3.0) | 7.2 (3.4) | H = 13.45 | **0.004** |
| Respiratory (mean, SD) | 10.1 (2.5) | 11.5 (1.0) | 11.3 (0.9) | 10.8 (1.2) | 6.9 (3.1) | H = 46.68 | **<0.001** |
| Bulbar (mean, SD) | 9.0 (3.1) | 9.8 (2.9) | 10.6 (2.5) | 8.8 (2.2) | 7.1 (3.6) | H = 23.76 | **<0.001** |

Furthermore, post hoc Dunn test explorations ALSFRS-R domains found those in Stage 4 had a significantly lower ALSFRS-R scores than those in Stage 1 across all domains, bulbar (p = .004), fine motor (p = .008), gross motor (p = .018) and respiratory (p < .001), as well as Stage 2 only on bulbar (p < .001) and respiratory (p < .001) domains. Additionally, those in Stage 4 had a significantly lower ALSFRS-R score than those in Stage 3 on only the respiratory (p < .001) domains. Stage 3 had significantly lower ALSFRS-R scores than those in Stage 1 across bulbar (p = .047) and gross motor (p = .002) domains, as well as Stage 2 only on the bulbar domain (p = .002). Those in Stage 2 had significantly lower scores than those in Stage 1 on fine motor (p = .009) and gross motor domains (p = .015).
