# Supplementary material for: Self-perceived quality of life, cognitive and behavioural impairment in amyotrophic lateral sclerosis
Source: J Neurol. 2024 Aug 28;271(10):6822–38. doi: 10.1007/s00415-024-12639-z (PMC11446980; doi:10.1007/s00415-024-12639-z)
Supplement: Supplementary file 2 — Supplementary file2 (DOCX 15 KB) [file 415_2024_12639_MOESM2_ESM.docx]

Online Resource 2.

ALSsQoL and subdomains for pwALS, subdivided and compared by Strong et al. (2017) diagnostic criteria (ALSci, ALSbi, ALScbi, ALSni)

|  | **All**  **(N= 121)** | **ALSni**  **(N= 47)** | **ALSci**  **(N= 35)** | **ALSbi**  **(N= 17)** | **ALScbi**  **(N= 22)** | **Statistic** | ***p* value** |
| --- | --- | --- | --- | --- | --- | --- | --- |
| ALSsQoL (mean, SD) /10 |  |  |  |  |  |  |  |
| Overall | 6.8 (1.3) | 6.9 (1.2) | 7.3 (1.3) | 6.0 (1.2) | 6.8 (1.1) | F = 4.14 | **0.008** |
| Negative Emotions | 6.8 (2.6) | 6.9 (2.4) | 7.8 (2.4) | 5.0 (2.3) | 6.9 (2.6) | H = 13.15 | **0.004** |
| Interaction | 8.5 (1.5) | 8.8 (1.3) | 8.9 (1.2) | 7.5 (1.5) | 8.0 (1.7) | H = 16.92 | **<0.001** |
| Intimacy | 5.4 (3.1) | 6.2 (3.0) | 5.6 (3.3) | 5.6 (2.0) | 3.6 (2.8) | H = 11.57 | **0.009** |
| Religiosity | 6.3 (3.7) | 5.6 (3.7) | 7.2 (3.7) | 5.7 (3.4) | 6.6 (4.1) | H = 5.27 | 0.15 |
| Physical Functioning | 6.4 (2.2) | 6.1 (2.4) | 6.7 (1.9) | 5.8 (2.2) | 7.3 (1.5) | H = 4.72 | 0.19 |
| Bulbar Function | 6.9 (3.0) | 6.6 (3.4) | 7.2 (2.7) | 6.9 (3.0) | 7.2 (2.6) | H = 0.61 | 0.90 |

ALS= Amyotrophic lateral sclerosis Specific Quality of Life Instrument; N= Number; SD= Standard Deviation; ALSni= ALS not impaired; ALSci= ALS cognitive impairment; ALSbi= ALS behavioural impairment; ALScbi= ALS cognitive and behavioural impairment
