# Supplementary material for: Self-perceived quality of life, cognitive and behavioural impairment in amyotrophic lateral sclerosis
Source: J Neurol. 2024 Aug 28;271(10):6822–38. doi: 10.1007/s00415-024-12639-z (PMC11446980; doi:10.1007/s00415-024-12639-z)
Supplement: Supplementary file 3 — Supplementary file3 (DOCX 18 KB) [file 415_2024_12639_MOESM3_ESM.docx]

Online Resource 3.

Hierarchical stepwise multiple regression results with ALSsQoL-SF bulbar function subdomain score as an outcome

|  | *b* | *β [95% CI]* | *p* | *R^2^* |
| --- | --- | --- | --- | --- |
| Step 1 |  |  |  | .062 |
| Age | -0.04 | -0.12 [-0.17, -0.06] | .20 |  |
| Gender | 0.90 | 0.14 -0.99, 1.28] | .11 |  |
| ALSFRS-R | 0.07 | 0.18 [0.10, 0.25] | .052 |  |
| Step 2 |  |  |  | .108 |
| Age | -0.03 | -0.11 [-0.16, -0.07] | .22 |  |
| Gender | 0.84 | 0.13 [-1.00, 1.27] | .15 |  |
| **ALSFRS-R** | **0.09** | **0.22 [0.14, 0.30]** | **.02** |  |
| Specific cognitive | -0.05 | -0.19 [-0.25, -0.14] | .10 |  |
| **Non-specific cognitive** | **0.14** | **0.22 [0.08, 0.37]** | **.045** |  |
| Behaviour | 0.07 | 0.04 [-0.29, 0.38] | .67 |  |
| Psychosis | 0.43 | 0.07 [-1.26, 1.39] | .51 |  |
| Step 3 |  |  |  | .115 |
| Age | -0.03 | -0.11 [-0.17, -0.06] | .22 |  |
| Gender | 0.84 | 0.13 [-1.02, 1.28] | .15 |  |
| **ALSFRS-R** | **0.08** | **0.18 [0.11, 0.26]** | **.047** |  |
| Memory | 0.03 | 0.04 [-0.10, 0.17] | .071 |  |
| Visuospatial | 0.38 | 0.12 [-0.48, 0.71] | .21 |  |

Significant p values are in **bold**. *b* = unstandardised beta; *β* = standardised beta; CI = confidence interval; ALSFRS-R= ALS Functional Rating Scale Revised

Hierarchical stepwise multiple regression results with ALSsQoL-SF physical function subdomain score as an outcome

|  | *b* | *β [95% CI]* | *p* | *R^2^* |
| --- | --- | --- | --- | --- |
| Step 1 |  |  |  | .067 |
| Age | -0.001 | -0.006 [-0.05, 0.03] | .95 |  |
| Gender | 0.03 | 0.007 [-0.81, 0.83] | .93 |  |
| **ALSFRS-R** | **0.08** | **0.26 [0.20, 0.31]** | **.005** |  |
| Step 2 |  |  |  | .104 |
| Age | -0.005 | -0.02 [-0.06, 0.02] | .81 |  |
| Gender | -0.06 | -0.01 [-0.84, 0.81] | .88 |  |
| **ALSFRS-R** | **0.08** | **0.25 [0.19, 0.31]** | **.008** |  |
| Specific cognitive | -0.02 | -0.13 [-0.17, -0.09] | .27 |  |
| Non-specific cognitive | -0.01 | -0.03 [-0.13, 0.08] | .81 |  |
| Behaviour | 0.14 | 0.12 [-0.13, 0.36] | .27 |  |
| Psychosis | -0.48 | -0.10 [-1.06, 0.86] | .32 |  |

Significant p values are in **bold**. *b* = unstandardised beta; *β* = standardised beta; CI = confidence interval; ALSFRS-R= ALS Functional Rating Scale Revised

Hierarchical stepwise multiple regression results with ALSsQoL-SF religiosity subdomain score as an outcome

|  | *b* | *β [95% CI]* | *p* | *R^2^* |
| --- | --- | --- | --- | --- |
| Step 1 |  |  |  | .110 |
| **Age** | **0.10** | **0.26 [0.20, 0.33]** | **.003** |  |
| **Gender** | **-1.50** | **-0.20 [-1.57, 1.20]** | **.03** |  |
| ALSFRS-R | 0.01 | 0.02 [-0.07, 0.11] | .84 |  |
| Step 2 |  |  |  | .122 |
| **Age** | **0.10** | **0.25 [0.18, 0.32]** | **.006** |  |
| **Gender** | **-1.58** | **-0.20 [-1.61, 1.21]** | **.03** |  |
| ALSFRS-R | 0.01 | 0.02 [-0.08, 0.12] | .82 |  |
| Specific cognitive | -0.01 | -0.05 [-0.11, 0.02] | .69 |  |
| Non-specific cognitive | -0.05 | -0.05 [-0.24, 0.13] | .62 |  |
| Behaviour | 0.02 | 0.009 [-0.41, 0.42] | .93 |  |
| Psychosis | 0.36 | 0.04 [-1.60, 1.69] | .66 |  |

Significant p values are in **bold**. *b* = unstandardised beta; *β* = standardised beta; CI = confidence interval; ALSFRS-R= ALS Functional Rating Scale Revised
